# Supplementary material for: Thinking clearly about misinformation
Source: Commun Psychol. 2024 Jan 5;2:4. doi: 10.1038/s44271-023-00054-5 (PMC11332083; doi:10.1038/s44271-023-00054-5)
Supplement: Supplementary file 1 — Supplementary Information [file 44271_2023_54_MOESM1_ESM.pdf]

Online Supplement to Tay et al. (2023). Thinking Clearly About Misinformation.

Supplementary Table 1

Examples of Misinformation Types

Type	Example
Systemic Lies	In the lead up to and during the 2003 invasion of Iraq, senior government officials peddled the narrative that Iraq held weapons of mass destruction that threatened the U.S. Misleading reports based on shoddy evidence were also produced in an attempt to link Saddam Hussein to Sep 11 (Calabrese, 2005). In a similar vein, vested-interest groups and fossil-fuel corporations have long sought to undermine effective climate action through the funding of contrarian scientists and conservative think tanks, and by engaging public-relations companies. Efforts have involved promoting cherry-picked data, generating uncertainty, and attacking the integrity of climate scientists (Boussalis & Coan, 2016; Dunlap & Brulle, 2020; Lewandowsky, 2021; Oreskes & Conway, 2010).
Truthiness	Media personalities such as Sean Hannity often aim to build personal connections with target audiences, which are then leveraged as emotional appeals for truth that sidestep the need for reasoned arguments and evidence (McCright & Dunlop, 2017). Arguably, social-media influencers on TikTok and Instagram also fall under this category, particularly when they take on undisclosed sponsorship of products (see also Kim et al., 2021). Such practices can range from innocuous to dangerous (e.g., undermining public-health responses and promoting supplements that are harmful; Bursztyn et al., 2020; MacFarlane et al., 2020).
Shock & Chaos	Russian disinformation campaigns often involve large volumes of content to foster doubt, sow confusion, and destabilize Western democracies. For example, when a Russian-made missile downed flight MH17 in 2014, Russia responded by denying the missile's origin, claiming the pilot had deliberately crashed the plane, or that Ukraine was to blame (e.g., Lewandowsky & Lynam, 2018). Such campaigns can also include the use of automated "bots" (e.g., Broniatowski et al., 2018; White, 2016), which can flood the landscape with false information and serve as links to questionable sources, exploiting algorithms that seek to maximize user engagement and making access to accurate information overall more difficult.
Bullshit	Purveyors of bullshit will spread any information seen as conducive to their cause without regard for truth. This includes headlines such as "Pfizer's Puppet President Biden Gives $9 Billion Taxpayer Funds for Millions More COVID Vaccines that Nobody Wants" and "Democrat Senator Ginny Talia was […] overheard saying: Americans over 70 should be euthanized" (O'Rourke, 2020). Journalists have also documented the litany of blatant falsehoods espoused by

	Trump on a range of topics (e.g., Kessler et al., 2019).
Paltering	Paltering is common across politics, marketing, espionage, and ordinary interactions. For example, during World War II, Nazi propagandist Joseph Goebbels used the fact that there was misinformation about Germany during World War I to discredit contemporary claims of German war crimes against the Jewish population (Marlin, 2013; Van Alstyne, 2022). Likewise, when the journalist Jim Lehrer asked Bill Clinton if he had a sexual relationship with his intern, Monica Lewinsky, Clinton responded with "There *is* not a sexual relationship – that is accurate". This response was technically true, as the relationship had ended, but was made with the intent to mislead (Rogers et al., 2014). Recent research has identified relatively high prevalence of misinformation in visual formats, including memes with misleading texts and images that place facts in misleading contexts (Yang et al., 2023). In addition, paltering can be inadvertent. For example, the American Diabetes Association's webpage on "Diabetes Myths" includes information that may be false only due to uncharitable reading. The labelling of such information as myths, although technically true, has been shown to lower readers' knowledge about diabetes (Powell et al., 2020).

Supplementary References

Boussalis, C., & Coan, T. G. (2016). Text-mining the signals of climate change doubt. *Global Environmental Change*, 36, 89-100. https://doi.org/10.1016/j.gloenvcha.2015.12.001

Broniatowski, D. A., Jamison, A. M., Qi, S., AlKulaib, L., Chen, T., Benton, A., Quinn, S. C., & Dredze, M. (2018). Weaponized Health Communication: Twitter Bots and Russian Trolls Amplify the Vaccine Debate. *American Journal of Public Health, 108*(10), 1378–1384. https://doi.org/10.2105/AJPH.2018.304567

Bursztyn, L., Rao, A., Roth, C. P., & Yanagizawa-Drott, D. H. (2020). *Misinformation During a Pandemic* (Working Paper No. 27417). National Bureau of Economic Research. https://doi.org/10.3386/w27417

Calabrese, A. (2005). Casus Belli: U.S. Media and the Justification of the Iraq War. *Television & New Media*, *6*(2), 153–175. https://doi.org/10.1177/1527476404273952

Dunlap, R. E., & Brulle, R. J. (2020). Sources and amplifiers of climate change denial. *Research Handbook on Communicating Climate Change*, 49–61.

Kessler, G., Rizzo, S., & Kelly, M. (2019). Analysis | President Trump has made more than 10,000 false or misleading claims. *Washington Post*. Retrieved July 27, 2022, from https://www.washingtonpost.com/politics/2019/04/29/president-trump-has-made-more-than-false-or-misleading-claims/

Kim, D. Y., & Kim, H.-Y. (2021). Trust me, trust me not: A nuanced view of influencer marketing on social media. *Journal of Business Research, 134*, 223–232. https://doi.org/10.1016/j.jbusres.2021.05.024

Lewandowsky, S. (2021). Climate Change Disinformation and How to Combat It. *Annual Review of Public Health*, *42*(1), 1–21. https://doi.org/10.1146/annurev-publhealth-090419-102409

Lewandowsky, S., Lynam, J. (2018). *Combating 'fake news': The 21st century civic duty*. The Irish Times. Retrieved November 17, 2022, from https://www.irishtimes.com/opinion/combating-fake-news-the-21st-century-civic-duty-1.3739327

MacFarlane, D., Tay, L. Q., Hurlstone, M. J., & Ecker, U. K. H. (2021). Refuting Spurious COVID-19 Treatment Claims Reduces Demand and Misinformation Sharing. *Journal of Applied Research in Memory and Cognition*, *10*(2), 248–258. https://doi.org/10.1016/j.jarmac.2020.12.005

Marlin, R. (2013). Propaganda and the Ethics of Persuasion. *Broadview Press.*

McCright, A. M., & Dunlap, R. E. (2017). Combatting misinformation requires recognizing its types and the factors that facilitate its spread and resonance. *Journal of Applied Research in Memory and Cognition, 6*(4), 389–396. https://doi.org/10.1016/j.jarmac.2017.09.005

O'Rourke, C. (2020). PolitiFact—No, Democrats aren't advocating euthanizing senior citizens to save Social Security. @politifact. Retrieved November 17, 2022, from https://www.politifact.com/factchecks/2020/may/14/blog-posting/no-democrats-arent-advocating-euthanizing-senior-c/

Oreskes, N., & Conway, E. M. (2010). Defeating the merchants of doubt. *Nature*, *465*(7299), 686–687. https://doi.org/10.1038/465686a

Powell, D., Bian, L., & Markman, E. M. (2020). When intents to educate can misinform: Inadvertent paltering through violations of communicative norms. *PLOS ONE*, *15*(5), e0230360. https://doi.org/10.1371/journal.pone.0230360

Rogers, T., Zeckhauser, R., Gino, F., Norton, M. I., & Schweitzer, M. E. (2017). Artful paltering: The risks and rewards of using truthful statements to mislead others.

Journal of personality and social psychology, 112(3), 456. https://doi.org/10.1037/pspi0000081

Van Alstyne, M. (2021*). Free Speech, Platforms & The Fake News Problem* (SSRN Scholarly Paper No. 3997980). https://doi.org/10.2139/ssrn.3997980

White, J. (2016, May). *Dismiss, Distort, Distract, and Dismay: Continuity and Change in Russian Disinformation IES Policy Brief Issue 2016/13•May 2016* [Policy Paper]. http://www.ies.be/policy-brief/dismiss-distort-distract-and-dismay-continuity-and-change-russian-disinformation

Yang, Y., Davis, T., & Hindman, M. (2023). Visual misinformation on Facebook. *Journal of Communication*, jqac051. https://doi.org/10.1093/joc/jqac051
